# Verifying the Placement of Nasogastric Tubes at an Emergency Center: Comparison of Ultrasound with Chest Radiograph

**DOI:** 10.1155/2018/2370426

**Published:** 2018-12-18

**Authors:** Çağdaş Yıldırım, Selçuk Coşkun, Şervan Gökhan, Gül Pamukçu Günaydın, Ayhan Özhasenekler, Uğur Özkula

**Affiliations:** ^1^Department of Emergency Medicine, Ankara Yıldırım Beyazıt University Medical School, Ankara 06800, Turkey; ^2^Department of Emergency Medicine, Ankara Atatürk Training and Research Hospital, Ankara 06800, Turkey; ^3^Department of Emergency Medicine, Girne University Dr. Suat Günsel Hospital, Girne 99320, KKTC, Cyprus

## Abstract

The objective of this study was to verify the nasogastric tube position with neck ultrasound and subxiphoid ultrasound, by giving air-water mixture and auscultation and to compare the effectiveness of these methods with chest radiography. This is a single-center, prospective, single-blind study. Patients who were admitted to our emergency department and had an indication of nasogastric tube placement were included. Nasogastric tube localization was verified with neck ultrasound and subxiphoid ultrasound, by giving air-water mixture, auscultation, and direct radiography that was accepted as the ‘gold standard technique'. A total of 49 patients (27 Male, 22 Female) with a mean age of 58.3±22.7 years were included. Sensitivity of neck ultrasound was 91.5%, and positive predictive value was 100%. As for the subxiphoid ultrasound sensitivity was 78.72%. When neck ultrasound + subxiphoid ultrasound and giving water-air mixture were combined sensitivity reached 95.74%. Sensitivity of neck ultrasound + subxiphoid ultrasound + air-water mixture + auscultation was 97.87% and positive predictive value was 100%. In the light of our results, neck and subxiphoid ultrasound seem to be an alternative method for verifying nasogastric tube localization. Combination of the air-water mixture and auscultation with ultrasound improves the sensitivity.

## 1. Introduction

Rapid and safe placement of the nasogastric (NG) tube is one of the most common and life-saving procedures in emergency department (ED) [1]. Although NG tube application is generally considered safe, it can result in complications such as pneumothorax, pneumomediastinum, subcutaneous emphysema, pneumonia, pulmonary hemorrhage, empyema, hemothorax, mediastinitis, bronchopleural fistula, perforation [2]. NG tube misplacement has been reported in quite different frequencies: 1.9-89.5% in adults and 20.9-43.5% in children in medical literature [3]. Therefore, it is necessary to verify correct placement in ED.

There are various methods that can be used to verify the location of the NG tube such as auscultation, direct chest radiography, pH measurement, calorimetric capnography, and ultrasound (US). [4–7] The ‘gold standard' method is radiation-containing (X-ray, computed tomography) confirmation. On the other hand, there has been growing utilization of US for verifying NG placement due to several advantages (widely available, easy applicable, provides repetitive evaluations, bedside evaluation, fast, cheap, lack of ionizing radiation, high spatial resolution, and provides dynamic imaging). Concerning the verification of NG tube placement with US, there is limited number of studies in the literature [4, 8–10]. These studies address ultrasonography as a promising method for NG tube verification with high sensitivity and specificity but this technique is not considered gold standard yet. Tian L. et al. reviewed five studies on diagnostic accuracy of US for detecting NG tube and they have found that sensitivity and specificity of US was 93% and 97%, respectively [11]. Accordingly, the objective of this study is to compare US with direct radiograph in verifying NG tube placement.

## 2. Materials and Methods

### 2.1. Study Design and Participants

This is a single-center, prospective, single-blind study. The study was conducted in an urban hospital's emergency medicine department with 150000 patient visits a year. Study protocol was approved by the Local Ethics Committee and was conducted in accordance with Helsinki Declaration.

Patients who were admitted to our ED with any complaint and had any indication of NG tube placement, between 01.02.2016 and 10.06.2016, were included in this study after their informed consent was obtained.

Inclusion criteria were as follows:

(1) To be older than 18 years

(2) Indication of NG tube placement between the dates mentioned and admitted to our adult ED

Indications used for NG tube placement during study period are as follows:

(i) Stomach decompression

(ii) Reducing the risk of vomiting and its incidence

(iii) Observing and evaluating the upper gastrointestinal bleeding risk

(iv) Prolonged ileus

(v) To give medication or oral contrast to nonswallowing patients

(vi) Detection of transdiaphragmatic herniation

(vii) Stomach lavage

(3) Voluntarily accepting participation in the work

Exclusion criteria were as follows:

(1) Midfacial injuries and head-base fractures

(2) Coagulopathy

(3) Stomach cuff or gastric by-pass

(4) Esophageal strictures or alkaline injury

(5) Patients who do not wish to participate in the study

(6) Patients who have open wound in the area to prevent ultrasonography and may be at risk of infection

**Figure 1:**
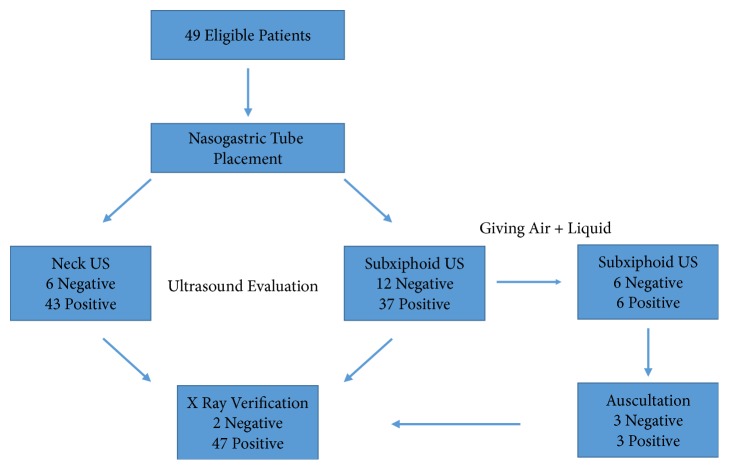
Flowchart.

According to these criteria patients were included and excluded from the study and the study flow is displayed in Figure 1.

### 2.2. Nasogastric Tube Placement, Ultrasonographic, and Radiographic Verification

**Figure 2:**
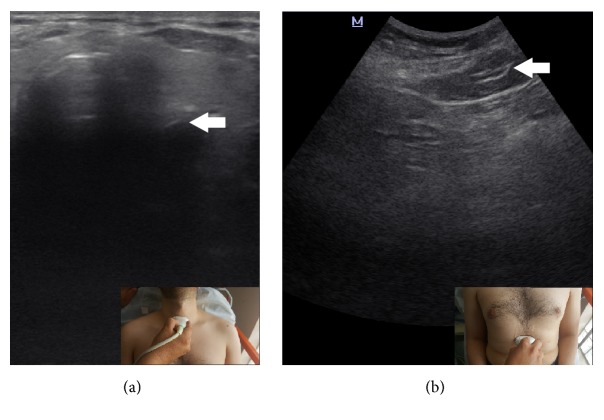
Neck ultrasound (a) and abdomen ultrasound (b) images show the nasogastric localizations. Small images denote the probe orientation.

The NG tube was placed by an emergency medicine resident who was primarily responsible for patient's management in the ED. NG tube length was determined by measuring the distance starting from to the tip of the nose to the tip of the patient's ear lobe and then to the xiphoid process and adding 10 cm to that measurement. The size of the NG tubes was determined as 16 Fr (standardized). After insertion of the tube, US evaluation was performed. All patients were evaluated by the same physician who was certified in ‘bedside ultrasonographic evaluation in emergency care'. Mindray (M5, Hamburg, Germany) US machine was used. A 7.5 MHz linear probe was used for neck visualization at the level of cricoid membrane (Figure 2(a)). A 3.5 MHz convex probe was used to visualize subxiphoid and gastroesophageal region (Figure 2(b)). If the NG tube could not be verified at the subxiphoid region, 10 cc air and 40 cc liquid mix was given with a pine-tipped syringe. Then, US evaluation was repeated. US performer should look for dynamic fogging in the stomach at the tip of the NG tube [4]. After that, the tube place was checked by auscultation method by injecting 10-20 cc of air with a pine-tipped syringe. Lastly, all patients were screened by direct radiography. Chest X-ray was accepted as the “gold standard” technique.

### 2.3. Statistical Analysis

IBM SPSS Statistics 21.0 (IBM Corp. released 2012. IBM SPSS Statistics for Windows, version 21.0, Armonk, NY) and MS-Excel 2007 programs were used for statistical analysis/calculations. Mean ± standard deviation was used with the median (minimum, maximum) for the descriptive statistics for the age variable. Number (n) and percent (%) values were given for the categorical variables such as gender, indications, and Glasgow Coma Scores. Sensitivity, specificity, positive predictive value (PPV), and negative predictive value (NPV) were calculated for each method.

## 3. Results

A total of 49 patients (27 Male, 22 Female) with a mean age of 58.3±22.7 years were included. Clinical and demographical features are summarized in Table 1.

When neck US was compared with chest X-ray [Table 2]; both neck US and X-ray could not detect NG tube in two patients. In these two patients, the NG catheter was folded in the pharynx. Direct radiograph showed the NG tube in 4 cases although neck US did not. In these cases, optimal imaging was not obtained with US from the neck region because of cachexia, subcutaneous emphysema, the patient's intolerance for US evaluation due to neck pain, and, in the last patient, patient's improper position for neck US due to the contractures. Confirmation of the last patient was made with subxiphoid US. There is no case that cannot be confirmed by plain radiograph and confirmed by neck US. Sensitivity of neck US was 91.49% and specificity was 100%, PPV was 100%, and NPV was 33.33%.

NG location could not be confirmed in 12 patients evaluated with subxiphoid US [Table 3]. Direct radiograph verified the tube in 10 patients. In two patients who could not be verified with X-ray, NG was folded in pharynx. There were no patients confirmed with subxiphoid US and could not be confirmed with X-ray. Sensitivity and specificity of subxiphoid US were 78.72% and 100%, respectively. PPV was 100% and NPV was 16.67%. When the subxiphoid US was repeated after the air-water mixture was given from the NG tube, the NG localization was confirmed in six patients [Table 4]. Sensitivity reached 91.49% and specificity was 100% with this technique. Positive and negative predictive values were 100% and 33.33%, respectively.

Sensitivity of neck US + subxiphoid US + air-water mixture combined was 95.74%, specificity was 100%, PPV was 100%, and NPV was 50% [Table 5]. Sensitivity of the neck US + subxiphoid US + air-water mixture + auscultation combined was 97.87%, specificity was 100%, and positive and negative predictive values were 100% and 66.67%, respectively. Overall, only one patient aside from the two patients whose tubes fold in the pharynx could not be checked with this technique. However, NG tube placement was confirmed with direct radiographs in this patient [Table 6].

## 4. Discussion

In this study we aimed to compare US with direct radiograph in verifying NG tube placement. According to our results, US is quite sensitive to detect right position compared with direct radiograph as a ‘gold standard technique'.

The current ‘gold standard' technique for verifying the location of the NG tube is direct chest X-ray. However, radiation exposure, cost, and relatively long time for administering seem to be the disadvantages [7]. Another method currently used for verification of tube placement is auscultation but the ‘rumbling' sound arising from the bronchial site is can be mistaken for the sound at epigastrium; thus it is not reliable. In a study conducted by Metheny et al. [12], it was concluded that auscultation was accurate in 34.4% of the cases to confirm NG placement.

Bedside US is another option that can be applied easily, without radiation, and it is fast. In a study conducted in 33 intensive care patients by Vigneauet al., NG tube placement with the guidance of US has been proven to be faster than direct chest X-ray and has 97% sensitivity [13]. NG tube with metal nose is used in the study, which is easy to fix and easy to detect by US because the metal is very hyperechoic and causes artifact (acoustic shadowing). However, in emergency departments, tubes produced entirely from PVC are widely used. Likewise, we also used a 16 Fr NG tube, which was made entirely from PVC and was not metallized and reached 95.74% sensitivity.

In our study, patients with a Glasgow Coma Score of 15 and no change in consciousness were included as well. Similar studies in the medical literature consisted of patients with altered level of consciousness [4] or intensive care patients [13, 14]. In our study both neck and subxiphoid US could not be performed in only one patient (the patient's Glasgow Coma Score was 15, it was a suicide-related emergency admission, with gastric lavage necessity) due to being not cooperative to procedure. Apart from that patient, we had 27 patients with a Glasgow Coma Score of 15 and US was done to all easily.

In our study, 43 (87.8%) of 49 patients were confirmed to have NG tube in the esophagus, with neck US. The NG tube was not seen in the esophagus in two patients since the tube was folded. In those patients the tube could not be visualized by plane radiograph, either. In a patient with cachexia, the reduction of subcutaneous fat tissue and the resulting level of sternocleidomastoid muscle between the sternal head and the trachea made ultrasound imaging impossible. Another patient in whom the tube could not be visualized had cerebral palsy and excessive neck contracture. As such, he could not be positioned. In these patients, NG location was confirmed with subxiphoid US. NG tube was verified with radiography in a patient who developed subcutaneous emphysema in the neck due to thorax trauma. US could not be performed in another patient because of lack of cooperation as well. These four patients were confirmed by direct chest X-ray. In a study conducted by Kim et al. [4], neck US was applied to 47 patients and 39 (83%) were verified with US. However, there was no discussion on why they could not see the NG tube in those 8 patients [4]. In another study done by Gök et al. [14] US was performed in 56 patients and the esophagus was detected with US before NG insertion. NG catheter was inserted in real-time with US. In three patients, the procedure was not performed with because the esophagus could not be detected [14]. In this study, the sensitivity for neck US is 98% while in our study it was 91.5%. Visualization of the tube in the esophagus by neck US only proves that the tube is in esophagus but visualization of the tube in the stomach is still required to prove correct positioning.

Nasogastric catheter location was confirmed in 37 (75.51%) of our patients who underwent subxiphoid US. NG catheter location was verified in 6 more patients after air-water mixture was given. When using subxiphoid US, sensitivity was 78.72%, while sensitivity reached 91.5% with the addition of air-water mixture. The most common challenges preventing visualizing during subxiphoid US was gas interposition due to ileus and perforation (n = 7). Likewise, in other studies performed with subxiphoid US, gas interposition is the most common reason as well [4]. US sensitivity was found to be 98.3% using only epigastric US in a study conducted by Chenaita et al. [15]. In another study, the sensitivity was 97% with air-water mixture [13]. In our study, sensitivity was only 78.72% with epigastric US and sensitivity reached 91.49% with air-water mixture. Since Vigneauet al. used a metal-tipped NG, it was thought that the sensitivity was 98%, unlike our study. In Chenaita et al. [15], emergency physicians performing US were described as having extensive experience in the field of US. These two facts might have affected our results.

NG catheter location could not be confirmed in four patients who underwent neck and subxiphoid US (including air-water mixture). In two of these patients the NG was folded in pharynx. In two other patients the NG tubes were not detected due to subcutaneous emphysema and the patients lack of cooperation for US exam. NG tube position was detected by direct chest X-ray in these two patients. Sensitivity was found to be 95.74%. Hyphen et al. [4] have found kappa value was 0.299 and sensitivity was 86.4% [4]. The difference may most likely be due to the fact that their study consisted of patients who had altered level of consciousness and the application of supine position.

Sensitivity reached 97.87% in our study when US was used together with auscultation verification. Previous studies did not reveal any statistical significance [4, 13–16].

We think that linear probe could be useful for subxiphoid view with pediatric and thin patients so procedure could be completed with only one probe.

After placement of NG tube we recommend readers to perform the subxiphoid US as first step. If the tube can be seen in the stomach then no other intervention is needed. If the subxiphoid US is negative then we recommend they perform neck US. If the tube can be seen in esophagus with neck US, then advance the tube and check with air-saline injection and perform subxiphoid US again and look for dynamic ‘fogging' to verify placement simultaneously. Lastly if the ultrasounds are positive, they are very predictive of good placement, but if negative, an X-ray should be done if possible prior to repositioning as the ultrasound can miss appropriately placed tubes. If X-ray is not available, then tube should be repositioned until ultrasound confirmed.

### 4.1. Limitations

We have a few important drawbacks for this study. First, our sample size could be larger. However, it is acceptable compared with the previous studies. Second, we have included the patients with high Glasgow Coma Scores. This fact might have affected the sensitivity.

### 4.2. Conclusion

In the light of our results, neck and subxiphoid US seem to be a method comparable to direct radiography in ED for verifying NG tube localization. Combination of giving air-water mixture and auscultation with US improves the sensitivity of US.

Accordingly, we highlight the role of US in verifying NG tube placement with several advantages in daily practice of emergency physicians. Further studies concerning the role of US in different patient groups (children, trauma, etc.) are awaited.

## Figures and Tables

**Table 1:** Clinical and demographical features.

**Variables**	**Data (n=49)**
**Age (mean±SD) year**	58.3±22.7
**Gender n (**%**)**	
(i) **M**	27 (55.1)
(ii) **F**	22 (44.9)
**NG Tube Indications n (**%**)**	
(i) **Ileus**	25 (51)
(ii) **Medication**	15 (30.6)
(iii) **Gastric Lavage**	5 (10.2)
(iv) **Gastrointestinal hemorrhage**	3 (6.1)
(v) **Perforation**	1 (2)
**Comorbidities n (**%**)**	
(i) **Yes / No**	23 (46.9) / 26 (53.1)
(a) **Atherosclerosis **	7 (14.2)
(b) **HT**	6 (12.2)
(c) **DM**	5 (10.2)
(d) **Congestive Heart Failure**	3 (6.1)
(e) **COPD**	2 (4.1)
(f) **Chronic Renal Failure**	3 (6.1)
(g) **Colon Cancer**	2 (4.1)
(h) **CVA**	3 (6.1)
(i) **Thrombocytosis**	1 (2.0)
(j) **Cerebral Palsy**	1 (2.0)
(k) **Sarcoidosis**	1 (2.0)
(l) **Alzheimer**	1 (2.0)
(m) **Schizophrenia**	1 (2.0)
**Glasgow Coma Scores n (**%**)**	
**<8**	10 (20.4)
**>8**	39 (79.6)

**M**, male; **F**, female; **HT**, hypertension; **DM**, diabetes mellitus; **COPD**, chronic obstructive pulmonary disease; **CVA**, cerebrovascular accident.

**Table 2:** Comparison of the neck ultrasound and direct radiograph.

		**Direct Radiograph**		**Total**	**Sensitivity**	**Specificity**	**PPV**	**NPV**
		**Negative**	**Positive**					
**Neck US**	**Negative**	2	4	6	91,49%	100%	100%	33.3%
	**Positive**	0	43	43				
**Total**		2	47	49				

**Table 3:** Comparison of the subxiphoid ultrasound and direct radiograph.

		**Direct Radiograph**		**Total**	**Sensitivity**	**Specificity**	**PPV**	**NPV**
		**Negative**	**Positive**					
**Subxiphoid US**	**Negative**	2	10	12	78.72%	100%	100%	16.67%
	**Positive**	0	37	37				
**Total**		2	47	49				

**Table 4:** Comparison of the subxiphoid ultrasound + air-water mixture with direct radiograph.

		**Direct Radiograph**		**Total**	**Sensitivity**	**Specificity**	**PPV**	**NPV**
		**Negative**	**Negative**					
**Subxiphoid US** **+** **Air-Water Mixture**	**Negative**	2	4	6	91.49%	100%	100%	33.33%
	**Positive**	0	43	43				
**Total**		2	47	49				

**Table 5:** Comparison of the neck ultrasound + subxiphoid ultrasound + air-water mixture with direct radiograph.

		**Direct Radiograph**		**Total**	**Sensitivity**	**Specificity**	**PPV**	**NPV**
		**Negative**	**Positive**					
**Neck US + Subxiphoid US + Air-Water Mixture**	**Negative**	2	2	4	95.74%	100%	100%	50%
	**Positive**	0	45	45				
**Total**		2	47	49				

**Table 6:** Comparison of the neck ultrasound + subxiphoid ultrasound + air-water mixture + auscultation with direct radiograph.

		**Direct Radiograph**		**Total**	**Sensitivity**	**Specificity**	**PPV**	**NPV**
		**Negative**	**Positive**					
**Neck US + Subxiphoid US + Air-Water + Auscultation**	**Negative**	2	1	3	97.87%	100%	100%	66.67%
	**Positive**	0	46	46				
**Total**		2	47	49				

## Data Availability

The data used to support the findings of this study are available from the corresponding author upon request.

## References

[1] Sorokin R., Gottlieb J. E. (2006). Enhancing Patient Safety During Feeding-Tube Insertion: A Review of More Than 2000 Insertions. *Journal of Parenteral and Enteral Nutrition*.

[2] Pillai J. B., Vegas A., Brister S. (2005). Thoracic complications of nasogastric tube: review of safe practice. *Interactive CardioVascular and Thoracic Surgery*.

[3] Ellett M. L. (2004). What is known about methods of correctly placing gastric tubes in adults and children. *Gastroenterology Nursing*.

[4] Kim H. M., So B. H., Jeong W. J., Choi S. M., Park K. N. (2012). The effectiveness of ultrasonography in verifying the placement of a nasogastric tube in patients with low consciousness at an emergency center. *Scandinavian Journal of Trauma, Resuscitation and Emergency Medicine *.

[5] Krauss B., Hess D. R. (2007). Capnography for Procedural Sedation and Analgesia in the Emergency Department. *Annals of Emergency Medicine*.

[6] Turgay A. S., Khorshid L. (2010). Effectiveness of the auscultatory and pH methods in predicting feeding tube placement. *Journal of Clinical Nursing*.

[7] O'Keefe S. J. D., Foody W., Gill S. (2003). Transnasal endoscopic placement of feeding tubes in the intensive care unit. *Journal of Parenteral and Enteral Nutrition*.

[8] Piton G., Parel R., Delabrousse E., Capellier G. (2017). Echography for nasogastric tube placement verification. *European Journal of Clinical Nutrition*.

[9] Dağlı R., Bayır H., Dadalı Y., Tokmak T. T., Erbesler Z. A. (2017). Role of ultrasonography in detecting the localisation of the nasoenteric tube. *Turk Anesteziyoloji ve Reanimasyon Dernegi Dergisi*.

[10] Zatelli M., Vezzali N. (2017). 4-Point ultrasonography to confirm the correct position of the nasogastric tube in 114 critically ill patients. *Journal of Ultrasound*.

[11] Lin T., Gifford W., Lan Y. (2017). Diagnostic accuracy of ultrasonography for detecting nasogastric tube (NGT) placement in adults: A systematic review and meta analysis. *International Journal of Nursing Studies*.

[12] Metheny N., McSweeney M., Wehrle M. A., Wiersema L. (1990). Effectiveness of the auscultatory method in predicting feeding tube location. *Nursing Research *.

[13] Vigneau C., Baudel J.-L., Guidet B., Offenstadt G., Maury E. (2005). Sonography as an alternative to radiography for nasogastric feeding tube location. *Intensive Care Medicine*.

[14] Gok F., Kilicaslan A., Yosunkaya A. (2015). Ultrasound-guided nasogastric feeding tube placement in critical care patients. *Nutrition in Clinical Practice*.

[15] Chenaitia H., Brun P.-M., Querellou E. (2012). Ultrasound to confirm gastric tube placement in prehospital management. *Resuscitation*.

[16] Nguyen L., Lewiss R. E., Drew J., Saul T. (2012). A novel approach to confirming nasogastric tube placement in the ED. *The American Journal of Emergency Medicine*.

